# Serum Testosterone Levels and Symptom-Based Depression Subtypes in Men

**DOI:** 10.3389/fpsyt.2015.00061

**Published:** 2015-05-04

**Authors:** Stephanie Rodgers, Martin grosse Holtforth, Michael P. Hengartner, Mario Müller, Aleksandra A. Aleksandrowicz, Wulf Rössler, Vladeta Ajdacic-Gross

**Affiliations:** ^1^Department of Psychiatry, Psychotherapy and Psychosomatics, Zurich University Hospital of Psychiatry, Zurich, Switzerland; ^2^Department of Psychology, University of Zurich, Zurich, Switzerland; ^3^Department of Psychology, University of Bern, Bern, Switzerland; ^4^Department of Applied Psychology, Zurich University of Applied Sciences, Zurich, Switzerland; ^5^Collegium Helveticum, Swiss Federal Institute of Technology, University of Zurich, Zurich, Switzerland; ^6^Laboratory of Neuroscience (LIM27), Institute of Psychiatry, University of São Paulo, São Paulo, Brazil

**Keywords:** testosterone, depression, subtypes, epidemiology, cross-sectional study

## Abstract

The main objective of this preliminary study was to further clarify the association between testosterone (T) levels and depression by investigating symptom-based depression subtypes in a sample of 64 men. The data were taken from the ZInEP epidemiology survey. Gonadal hormones of a melancholic (*n * = 25) and an atypical (*n* = 14) depression subtype, derived from latent class analysis, were compared with those of healthy controls (*n * = 18). Serum T was assayed using an enzyme-linked immunosorbent assay procedure. Analysis of variance, analysis of covariance, non-parametrical tests, and generalized linear regression models were performed to examine group differences. The atypical depressive subtype showed significantly lower T levels compared with the melancholic depressives. While accumulative evidence indicates that, beyond psychosocial characteristics, the melancholic and atypical depressive subtypes are also distinguishable by biological correlates, the current study expanded this knowledge to include gonadal hormones. Further longitudinal research is warranted to disclose causality by linking the multiple processes in pathogenesis of depression.

## Introduction

Apart from its gonadal functions, testosterone (T) has a significant influence on the human brain through various neurobiological processes (1–3). In men, associations between T levels and depressive symptoms have been proposed (3, 4). Such neuroendocrine dysfunctions may play an important role in the pathogenesis of major depressive disorder (MDD) (5). In particular, low T levels (hypogonadism) have been associated with depression (4, 6–8), and there is evidence that T secretion is impaired during depressed mood (9, 10). However, this association has not been observed consistently and some studies did not find any relationship between T levels and depression (11, 12). These inconsistent findings could be explained by the heterogeneity of the construct of MDD (13) or difficulties concerning the measurement of salivary T versus that of serum T (14). Moreover, many findings stem from studies involving exogenous T administration, whereas studies looking at the effects of endogenous T are lacking (15). In sum, there appears to be insufficient evidence to conclude that low T levels are routinely involved in the pathogenesis of MDD in men (15).

However, a growing body of evidence suggests that there may be subpopulations of men vulnerable to depression in which hypogonadism is a contributing factor (16). Some studies showed that low T levels were specifically associated with subthreshold depressive disorders, such as dysthymia or minor depression (15, 17–20). Other studies demonstrated that hypogonadism was related to the specific depressive symptoms of dysphoria, irritability, fatigue, lethargy, decreased libido, and decreased concentration (6, 15). This symptom cluster has also been conceptualized as “irritable male syndrome,” occurring in adult male mammals following withdrawal of T (21).

Meanwhile, some investigations have drawn attention to non-linear associations between total T levels and depressive symptoms, with increased depression rates at both the lowest and the highest extremes of T levels (15, 22). Furthermore, exogenous T administration has not only been related to depression but also to hypomania and even mania (23–28). Despite these suggestive findings, to date, the associations between dysregulated endogenous T levels and affective symptoms remain fairly unclear (15).

Apart from the findings with regard to the hypothalamic– pituitary–gonadal (HPG) axis, previous studies have investigated associations between atypical and melancholic (typical) depression and the hypothalamic–pituitary–adrenal axis (HPA), the inflammatory response system and metabolic abnormalities (29, 30). Melancholic depression has been characterized by hypercortisolemia (HPA axis), whereas atypical depression has been associated with the opposite imbalance, namely hypocortisolemia (31). The HPA axis also interacts with gonadal hormones (32, 33) and Sigurdsson et al. (34) found a positive correlation between evening cortisol and evening T levels. Therefore, one might hypothesize that melancholic depression is associated with high T levels and atypical depression shows the opposite, that is to say low T levels. Moreover, T levels are negatively correlated with the body mass index (BMI) (35), and atypical depression is characterized by a higher BMI (36), further supporting the hypothesis. Consequently, the melancholic and atypical depression subtypes might not only be distinguishable by differing activity of the HPA axis but by that of the HPG axis as well.

In view of the inconsistency of the findings summarized above, this preliminary study set out to examine the differences in serum T levels in symptom-based depression subtypes, compared with healthy controls. The classification of these depression subtypes was based on a previous latent class analysis, derived from the depressive symptom profiles of an epidemiological sample of males (37). The purpose of this paper is to provide an alternative approach to the area of research by analyzing T levels in empirically derived depression subgroups. Based on previous findings with regard to T and depression and by drawing analogies to the HPA axis, we expected differing T levels between these depressive subgroups, with higher T levels for the melancholic depression subgroup and lower T levels for the high BMI associated, atypical depression subgroup.

## Materials and Methods

### Study design and sampling

**Figure 1:**
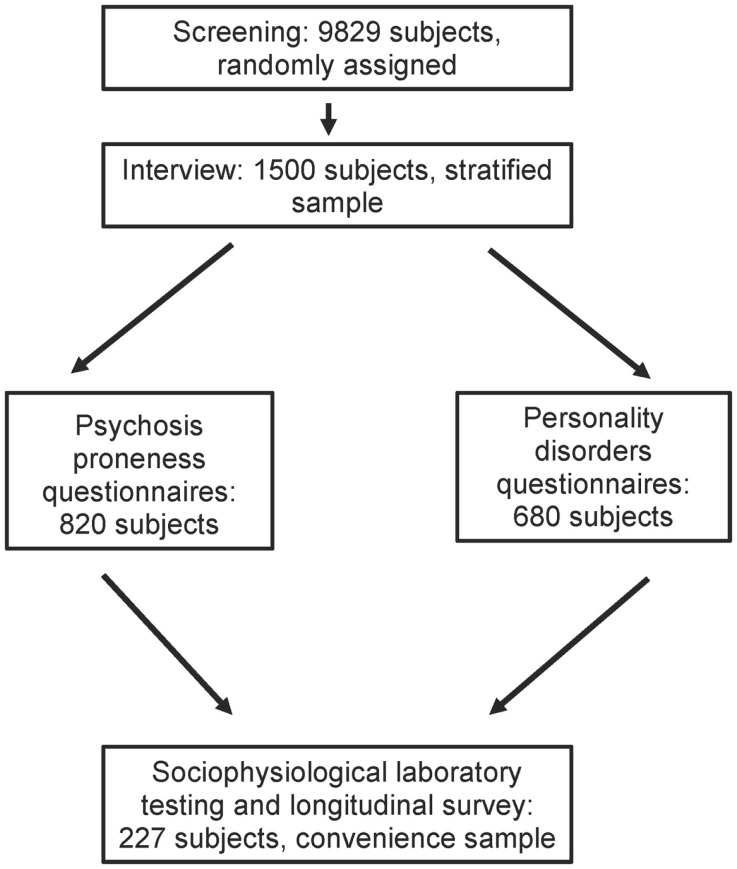
**The sampling procedure of the ZInEP epidemiology survey**.

Data from the epidemiology survey of the Zurich Program for Sustainable Development of Mental Health Services (ZInEP; German: *Zürcher Impulsprogramm zur nachhaltigen Entwicklung der Psychiatrie*) were used. The epidemiology survey was conducted in order to collect comprehensive data about mental health in the general population of adults in the canton of Zurich. The survey was methodologically designed as a cross-sectional sequel to the prospective Zurich cohort study (38), e.g., the instruments and the age structure were parallelized. It consisted of three components: (a) a brief telephone screening (*n * = 9829), (b) a structured face-to-face-interview supplemented by self-report questionnaires (*n * = 1500), and (c) a laboratory day followed by a longitudinal survey (*n * = 227) (see Figure 1). The survey was carried out between August 2010 and September 2012. In the following, only the laboratory day (c) providing the biological data will be elucidated. For more details with regard to (a) and (b), see Ajdacic-Gross et al. (39).

For the laboratory day, a subsample of 227 subjects was selected from the face-to-face sample (*n * = 1500). Sample construction was based on the outcomes of two psychoticism scales [schizophrenia nuclear symptoms scale (SNS) and schizotypal signs scale (STS) (40)], which were included in the screening interview, as follows: highest quintile on both scales; highest quintile on the SNS, any quintile on the STS; highest quintile on the STS, any quintile on the SNS; controls with a low overall global severity index (GSI) (41); and no use of professional help. The participation rate was 53.8% and depended mainly on the ease of acquiring the subjects for the time-consuming set of tests performed during a day in the sociophysiological laboratory of the Zurich University Hospital of Psychiatry. The participants were interviewed, provided saliva, blood, and urine samples, completed computer based tests, and underwent several tests involving electroencephalographic (EEG) and near infrared spectroscopy (NIRS) measurement. All tests were carried out by a biologist and three assistants (psychology students). Physicians from the Zurich University Hospital of Psychiatry were involved in the procedure of blood sampling. The gonadal hormones were exclusively derived from the blood probes. However, no gonadal hormone assays were taken from 37 subjects. The reasons for this were participants refusing blood sample collection or difficulties during blood sampling (e.g., problematic positions of veins). Of the 190 participants (men: *n * = 89; women: *n * = 101) with complete hormone data, 137 subjects (72.1%) were initially allocated to the stratum of high-scorers, while 53 subjects (27.9%) were low scorers.

The ZInEP epidemiology survey study was approved by the Ethical Committee of the canton of Zurich (KEK) and is in accordance with the latest version of the Declaration of Helsinki. After being extensively informed about study procedure and aims both verbally and in writing, the participants gave their written consent.

### Measurements

#### Depressive Symptom Subtypes

Depressive symptom subtypes were assessed by a computer-assisted version of the Structured Psychopathological Interview and Rating of the Social Consequences of Psychological Disturbances for Epidemiology (SPIKE) used in the Zurich Study (38, 42). This instrument includes the most common psychopathological syndromes/disorders. The validity and reliability of the SPIKE have been established for the assessment of depression and anxiety (38). For the current study, we made use of the three class solution, which had previously been extracted by a data-driven technique (latent class analysis, LCA) from the SPIKE data of 373 men manifesting depressive symptoms during the past 12 months (37): a melancholic depression subtype (*n* = 219; 58.7%), an atypical subtype (*n* = 64; 17.2%), and a moderate subtype (*n* = 90; 24.1%). We excluded the moderate subtype because the investigation of the two clear discriminable melancholic and atypical depression subtypes increased statistical power, which was necessary due to the small sample sizes. The melancholic and atypical depression subtypes are in line with previous subtyping approaches and/or the DSM-5 specifiers (36, 43–47). The distinction of these two subtypes on the basis of appetite-/weight related symptoms was particularly striking. Members of the atypical subtype showed high probabilities of increased appetite and weight gain, whereas the melancholic subtype was characterized by loss of appetite and weight. More detailed information about these depressive subtypes and the entire LCA procedures can be found elsewhere (37).

Overall, the T data were available for *n * = 89 men. From this group, 39 subjects either matched with the melancholic or the atypical depression subtype of the LCA three class solution (37). A subgroup of 18 controls (defined without use of professional help and low overall GSI-scores) was included. Consequently, the present study was dealing with a sample of 57 subjects.

#### Psychosocial Characteristics

In order to characterize the depression subtypes, sociodemographic variables such as age, education, urbanicity, and marital status were drawn from the face-to-face interview. Axis I diagnoses were computed according to DSM-III-R/IV criteria for the time-period of the past 12 months (48, 49). Only the diagnosis of neurasthenia was derived from ICD-10 (50). Due to methodological linkage with the Zurich Study (38), the algorithms of the DSM diagnoses were parallelized. As a consequence, some diagnoses were based on an older edition of the DSM. Psychosis syndromes were derived according to the algorithm of Rössler et al. (40); bipolar disorder was derived in analogy to the BRIDGE study (51). Personality disorders were assessed using the German translation of the assessment of DSM-IV personality disorders questionnaire (ADP-IV) (52, 53), showing good concordance with the SKID-II semi structured interview (54). The current smoking status and the GSI were derived from the CATI. Finally, the data concerning the consumption of psychiatric medication were taken from the laboratory day.

#### Biological Measures

##### Hormones

In order to ensure consistency due to diurnal variation (6) and in accordance with the recommendation of an earlier study (55), the blood sample was drawn in the morning. The exact time frame of the blood sample collection lay between 08:55 a.m. and 10:34 a.m., 01:45–03:51 h after the subject’s awakening following an overnight fast. Blood was centrifuged (10 min; 3000 rpm) 30 min after the sample was taken and stored at −80°C until delivered to the CYTOLAB for biochemical analysis. Total T was derived from serum and measured by enzyme-linked immunosorbent assay (ELISA) using a kit from IBL International, Hamburg, Germany. Dehydroepiandrosterone-sulfate (DHEA-S), luteinizing hormone (LH), estradiol, and progesterone were also derived from serum. While DHEA-S, estradiol, and progesterone were likewise assayed by ELISA (DHEA-S: IBL International, Hamburg, Germany; estradiol and progesterone: BioCheck, Foster City, USA), LH was measured by Bead-Assay (Millipore, Zug, Switzerland). Sex hormone-binding globulin (SHBG) was derived from ethylene diamine tetraacetic acid (EDTA) plasma and measured by ELISA using a kit from IBL International, Hamburg, Germany.

##### Blood pressure and BMI

Blood pressure was measured on the left upper arm in a sitting position at the beginning of the laboratory assessment and 15 min later with the Boso Medicus Prestige (Bosch + Sohn GmbH & Co, KG DE). Both systolic and diastolic values were obtained and averaged across the two measurements. BMI was conventionally calculated, based on mass and height (kg/m^2^).

#### Statistical Analysis

All data were analyzed using SPSS version 20 for Macintosh (IBM Corp., Armonk, NY, USA). The criterion for significance was set at *p* < 0.05.

First, Chi^2^-tests and Fisher exact tests (two-tailed) and non-parametric Kruskal–Wallis tests were performed in order to assess differences of psychosocial characteristics between the depressive subgroups atypical depression, melancholic depression, and controls.

Second, unadjusted analyses of variance (ANOVAs) were performed to evaluate the between-group differences on metric dependent variables. Before performing the ANOVAs, Levene’s tests were computed in order to examine the homogeneity of variances, and Shapiro–Wilk tests were used to account for the assumption of normally distributed residuals. The values of T were log transformed (natural logarithm, ln) due to the non-normally distributed residuals of the control group. In the ANOVAs, the depressive subgroup served as independent variable and the biological characteristics were treated as the dependent variable. Corresponding effect sizes were reported in terms of partial η^2^ [0.01 = small, 0.06 = medium, 0.14 = large; (56)]. In case of significant main effects, Scheffé and Games–Howell *post hoc* comparisons were used to explore all possible pairwise subgroup comparisons. Categorical biological characteristics were compared between the subgroups with the Fisher’s exact test for categorical variables (two-tailed). In addition, a non-parametric median test was carried out on the T data. Finally, an analysis of covariance (ANCOVA) considering the influence of the covariates age, smoking, alcohol use, and psychopharmacological medication was computed.

Third, the subgroup differences were further examined by generalized linear regression models (GLMs) using logistic regression. GLMs enable the use of robust estimators in order to minimize the effects of outliers and influential observations (57). T was entered as independent variable and the subgroups served as dependent variable.

## Results

### Psychosocial characteristics

In Table 1, the psychosocial characteristics of the LCA-based depressive subtypes and the control sample are displayed. No significant differences in demographic correlates were observed between these subsamples. However, groups differed with regard to some comorbid 12-month diagnoses. As expected, both depressive subtypes had higher frequencies of MDD diagnosis than the controls. The severe atypical subtype revealed a diagnosis of generalized anxiety disorder (GAD) and simple phobia significantly more often than the controls. In comparison with the melancholic subtype, agoraphobia occurred significantly more often in the atypical depression subtype. Atypical depression showed significantly more psychosis syndromes and personality disorders than the melancholic depression subtype and the controls. Antidepressant consumption was significantly associated with the atypical depression subtype (versus controls). Finally, in terms of the subjectively rated burden related to health problems, both depressive subtypes showed significantly higher values compared with controls.

**Table 1:** **Psychosocial characteristics for men belonging to the depressive subtypes derived from latent class analyses (*n* = 39) and healthy controls (*n* = 18), unadjusted**.

Characteristic^a^	Atypical MDD	Melancholic MDD	Controls	*p*-value
	*n * = 14	*n* = 25	*n * = 18	
				**Chi^2^/*F*-test (two-tailed, overall; *post hoc* tests comparing each subtype separately with all others)**
Age				0.957
21	4 (28.6)	3 (12.0)	3 (16.7)	
23	2 (14.3)	4 (16.0)	2 (11.1)	
28	3 (21.4)	6 (24.0)	5 (27.8)	
30	2 (14.3)	4 (16.0)	2 (11.1)	
35	2 (14.3)	2 (8.0)	3 (16.7)	
41	1 (7.1)	6 (24.0)	3 (16.7)	
Education^b^, *n* (%)				0.211
Low	11 (78.6)	16 (64.0)	8 (47.1)	
High	3 (21.4)	9 (36.0)	9 (52.9)	
Urbanicity^c^, *n* (%)				0.222
Urban	13 (92.9)	19 (76.0)	12 (66.7)	
Rural	1 (7.1)	6 (24.0)	6 (33.3)	
Marital status, *n* (%)				0.608
Unmarried	9 (64.3)	19 (76.0)	14 (77.8)	
Married	4 (28.6)	6 (24.0)	4 (22.2)	
Divorced	1 (7.1)	0 (0.0)	0 (0.0)	
Comorbidities^d^ (past year) *n* (%)				
MDD^e^	6 (42.9)	13 (52.0)	0 (0.0)	<0.001^r^^,^ ^s^
Dysthymia^e^	2 (14.3)	2 (8.0)	0 (0.0)	0.267
Hypomania/mania^f^	3 (21.4)	4 (16.0)	0 (0.0)	0.105
Bipolar disorder^g^	3 (21.4)	2 (8.0)	0 (0.0)	0.111
Neurasthenia^h^^,^ ^i^	1 (7.1)	6 (27.3)	1 (5.6)	0.144
GAD^j^	5 (35.7)	2 (8.0)	0 (0.0)	<0.01^r^
Simple phobia^f^	5 (41.7)	4 (19.0)	0 (0.0)	<0.05^r^
Social phobia^f^	3 (21.4)	5 (23.8)	0 (0.0)	0.061
Agoraphobia^f^	3 (21.4)	0 (0.0)	0 (0.0)	<0.05^q^
Psychosis syndromes^k^	5 (35.7)	1 (4.0)	0 (0.0)	<0.01^q^^,^ ^r^
Panic disorder^f^	1 (8.3)	2 (8.3)	0 (0.0)	0.443
OCD^f^	1 (7.1)	1 (4.0)	0 (0.0)	0.718
Binge eating^f^^,^ ^l^	4 (28.6)	2 (8.0)	1 (5.6)	0.144
Anorexia nervosa^e^	0 (0.0)	1 (4.0)	0 (0.0)	n/a
Suicide attempt	1 (10.0)	1 (5.9)	0 (0.0)	0.695
Alcohol dependence^f^	2 (14.3)	4 (16.0)	0 (0.0)	0.214
Alcohol abuse^f^	1 (7.1)	1 (4.0)	0 (0.0)	0.718
Current smoking	4 (28.6)	11 (44.0)	5 (27.8)	0.525
Any personality disorder^m^	4 (28.6)	1 (4.0)	0 (0.0)	<0.05^q^^,^ ^r^
Psychopharmaceuticals, *n*^n^	4 (30.8)	4 (23.5)	0 (0.0)	0.776
Antidepressant	4 (28.6)	2 (8.0)	0 (0.0)	<0.05^r^
Mood stabilizer	1 (7.1)	1 (4.0)	0 (0.0)	0.718
Sedative, hypnotic, anxiolytic	1 (7.1)	0 (0.0)	0 (0.0)	0.246
Neuroleptics	1 (7.1)	0 (0.0)	0 (0.0)	0.246
Other	1 (7.1)	1 (4.0)	0 (0.0)	0.718

Subjective impairment (mean ± SE)^o^				**Kruskal–Wallis test^p^**
GSI	2.25 (±0.16)	2.17 (±0.07)	1.34 (±0.06)	<0.001^r^^,^ ^s^

*MDD, major depressive disorder; DYST, dysthymia; GAD, generalized anxiety disorder; OCD, obsessive–compulsive disorder; n/a, not available*.

*^a^The discrepancy between the total number of persons and the number of persons in the following rows result from missing items*.

*^b^Although the Swiss educational degrees do not entirely correspond with the school system of Anglo-American areas or other countries, the educational degrees were comparatively grouped into the two following categories: low, less than high school diploma; high, high school diploma or higher*.

*^c^Urban: Zurich, Winterthur; rural: remaining municipalities of the canton of Zurich*.

*^d^12-month prevalence*.

*^e^DSM-III-R*.

*^f^DSM-IV*.

*^g^Def. BRIDGE study (51)*.

*^h^ICD-10*.

*^i^3-month criteria*.

*^j^DSM-III*.

*^k^Disorders of form of thought, derealization, depersonalization, delusion, disorder of ego-boundary, hallucinations, paranoia syndrome*.

*^l^Including binge eating symptoms*.

*^m^Paranoid, schizotypal, avoidant, dependent, obsessive–compulsive, borderline, histrionic, narcissistic, and depressive*.

*^n^Consumption of psychopharmaceuticals during the past 6 months*.

*^o^Global severity index (GSI) of the SCL-27 (41)*.

*^p^*Post hoc* tests: pairwise Kruskal–Wallis tests*.

*^q^Atypical MDD significantly differs from melancholic MDD*.

*^r^Atypical MDD significantly differs from controls*.

*^s^Melancholic MDD significantly differs from controls*.

### Biological characteristics

The biological characteristics of the subsamples are summarized in Table 2. The following T means and standard errors (SEs) (in ng/ml) were observed: atypical subtype: 3.92 (0.37), melancholic subtype 5.33 (0.28), and controls 4.87 (0.33). Log transformed data were used for computing the ANOVA. The atypical depression subtype differed significantly from the melancholic subtype with regard to lower T levels. The difference with respect to the controls remained on a trend level of significance. The same subgroup differences were found for SHBG. Furthermore, men manifesting atypical depressive symptom configurations were significantly more prone to a higher BMI than the melancholic depression subgroup and controls, respectively. The corresponding effect sizes of T, SHBG, and BMI were large.

**Table 2:** **Biological characteristics of men belonging to the depressive subtypes derived from latent class analyses (*n* = 39) and healthy controls (*n* = 18), unadjusted**.

Biological characteristics	Atypical MDD	Melancholic MDD	Controls	
	*n* = 14	*n* = 25	*n* = 18	
	Mean (SE)	Mean (SE)	Mean (SE)	Eta (η^2^)	*p*-value^a^
Testosterone (ln)	1.32 (0.08)	1.64 (0.06)	1.54 (0.07)	0.17	≤0.01^e^^,^ ^(f)^
SHBG (nmol/L)^b^	26.10 (4.29)	42.04 (2.71)	40.44 (4.52)	0.20	≤0.01^e^^,^ ^(f)^
DHEA-S (ug/mL)	2.17 (0.81)	2.48 (0.69)	2.31 (0.98)	0.02	0.513
LH (mlU/ml)^c^	2.59 (0.56)	3.18 (0.37)	2.80 (0.61)	0.02	0.653
Estradiol (ng/mL)	28.18 (2.10)	26.32 (1.57)	26.72 (1.85)	0.01	0.774
Progesterone (ng/mL)	0.87 (0.14)	1.01 (0.11)	0.92 (0.13)	0.01	0.713
BMI^d^	29.03 (0.92)	23.43 (0.67)	23.06 (0.78)	0.36	≤0.001^e^^,^ ^f^
Diastolic BP (mmHg)^d^	88.58 (2.98)	86.04 (2.15)	82.75 (2.53)	0.04	0.324
Systolic BP (mmHg)^d^	133.23 (3.51)	133.10 (2.53)	128.94 (2.99)	0.02	0.515

*MDD, major depressive disorder; ANOVA, analysis of variance; SHBG, sex hormone-binding globulin; DHEA-S, dehydroepiandrosterone-sulfate; LH, luteinizing hormone; BMI, body mass index; BP, blood pressure*.

*^a^One-way ANOVA/*post hoc* tests: Scheffé (homogeneous variances), Games–Howell (non-homogeneous variances)*.

*^b^Missing data: atypical MDD, *n * = 4; controls, *n * = 9*.

*^c^Missing data: atypical MDD, *n * = 3; controls, *n * = 9*.

*^d^Missing data: atypical MDD, *n * = 1*.

*^e^Atypical MDD significantly differs from melancholic MDD*.

^f^Atypical MDD significantly differs from controls

*^(f)^Parenthesis indicating trend-level associations *p* ≤ 0.12*.

In an extension of the analyses, the biological factors were categorized in order to validate the results in terms of medically relevant cut-offs: hypogonadism, which was defined as <2.3 ng/mL (58), occurred in *n * = 2 (14.3%) men belonging to the atypical depression subtype and the overall group differences could only be expressed as trend level associations (χ^2^ = 4.119, *df * = 2, *p * = 0.06). The categorized BMI (normal weight: BMI 18.50–25.00; overweight: BMI 25.01–30.00; obesity: ≥30.01) showed significant overall differences (χ^2^ = 12.435, *df * = 4, *p * < 0.05) with significantly differing frequencies between the atypical depression subtype [normal weight: *n * = 5 (38.5%); overweight: *n * = 4 (30.8%); obesity: *n * = 4 (30.8%)] and the melancholic depression subtype [normal weight: *n * = 21 (84.0%); overweight: *n * = 4 (16.0%); obesity: *n * = 0 (0.0%)], and controls [normal weight: *n * = 13 (72.2%); overweight: *n * = 5 (26.8%); obesity: *n * = 0 (0.0%)], respectively. The categorized blood pressure revealed no single cases of hypotension (defined as <90/60 mmHg). In contrast, hypertension (defined as >140/90 mmHg) occurred most frequently in the melancholic depression subtype (*n * = 8; 72.7%). However, the overall differences failed to achieve a customary level of statistical significance (χ^2^ = 4.539, *df * = 2, *p * = 0.107) (data not tabulated).

**Figure 2:**
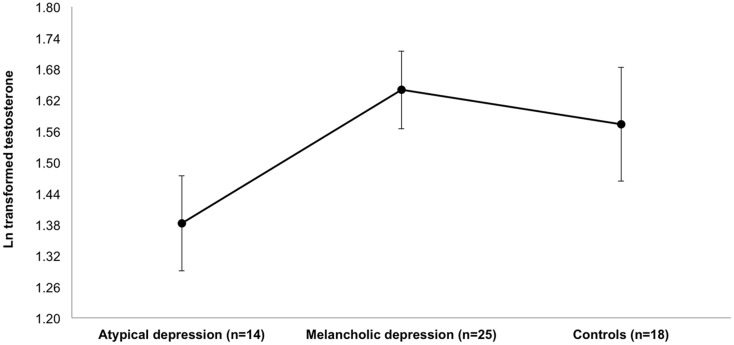
**Comparison of mean (±SE) log transformed (natural logs, ln) serum testosterone levels between the depressive subtypes and healthy controls after adjustment for age, smoking, alcohol abuse, and psychopharmacological medication**.

The non-parametric median test provided significant overall subgroup differences regarding the T levels (χ^2^ = 6.685, *df * = 2, *p * < 0.05). An additional ANCOVA, adjusted for the influence of age, smoking, alcohol use, and psychopharmacological medication, showed comparable results (*F* = 3.472, *df * = 2, *p * < 0.05). The BMI was neither included in the ANCOVA nor the following adjusted models because it showed high multicollinearity with the atypical depressive subtype and T, respectively. The adjusted means and SEs are visualized in Figure 2.

Generalized linear regression models were carried out by contrasting the three subgroups, while adjusting for age, smoking, alcohol use, and psychopharmacological medication. The analysis contrasting the atypical depression subgroup with the melancholic subgroup and controls, respectively, showed significant odds ratios (OR) between atypical and melancholic (reference) depression [OR = 0.024, 95% confidence intervals (CI): 0.001–0.640, *p* < 0.05] with lower T levels in the atypical subgroup. Apart from a trend-level association, T levels did not significantly differ between atypical depression and controls (reference) (OR = 0.103, CI: 0.007–1.576, *p* = 0.10). No significant difference was found between the T levels of the melancholic depressives and controls (reference) (OR = 2.806, CI: 0.190–41.375, *p* = 0.45) (data not tabulated).

## Discussion

This is a preliminary study examining differences of serum T levels in empirically derived symptom-based depression subtypes and a healthy control group in a Swiss community sample of male adults. The results provided evidence that the atypical depression subtype is discriminable by low serum T levels in men aged between 21 and 41. First, our results support the notion that the inconsistent evidence from previous studies with respect to T levels and MDD may have resulted from the heterogeneity of this diagnosis, while the investigation of more homogeneous symptom-based subtypes provides a more detailed picture. Second, the current analyses confirmed previous findings showing that the melancholic and atypical depressive subtypes were distinguishable by biological correlates (29, 31), and expanded this knowledge via the aspect of gonadal hormones.

Our results suggest that the previous findings of low T levels in depressed men (4, 6, 8–10) might have resulted from the subgroup of atypical depressives. In fact, the “irritable male syndrome,” a behavioral state following withdrawal of T in adult male mammals (21), shows a certain similarity to the symptoms of atypical depression (30) in human adults. There are several explanations for this specific biological correlate within atypical depressives. The low T levels in atypical depressives could be explained by the high BMI associated with this subtype; this marked biological characteristic of atypical depression will be discussed further below. A further conceivable analogy can be drawn from studies demonstrating differences in HPA axis regulation between melancholic and atypical depression (31, 59). Melancholic depression was characterized by hypercortisolism while atypical depression was associated with hypocortisolism (31). In combination with the evidence concerning the interactive nature in which the HPA and the HPG axes operate (33) this opens up interesting perspectives. A fairly recent community study on a sample of men showed a positive correlation between endogenous evening saliva T and overall depression scores, as well as evening cortisol (HPA axis) (34, 60). This relation was explained by the effect of cortisol modifying the gonadotropin-releasing hormone (GnRH) from the hypothalamus, by influencing the LH from the pituitary or by alterations in the stimulating effect of gonadotropins or gonads (34, 61). To return to the depression subtypes, it thus appears plausible that the down-regulated HPA axis is correlated with low T levels, or even hypogonadism, in atypical depressives. Also, SHBG, which is correlated with T (35), was significantly lower in the atypical depression subtype compared with the melancholic subtype. In line with this hypothesis, the hypercortisolism-associated melancholic subtype should show the opposite, namely T level alterations.

The initially expected higher T levels in the melancholic depression subtype were based on the available literature concerning the HPA axis dysregulation of this subtype (31) and exogenous T administration demonstrating that a high T dose resulted in both depression and hypomania/mania (24–28). In our study, T levels were actually higher in the melancholic depression subtype compared to both other groups, although they only differed significantly from the atypical group. Therefore, our two depression subtypes were found at both the lowest and highest areas of T distribution, which would be in line with the parabolic model displaying a curvilinear relationship between T and depression (13, 15, 22). However, considering the fact that the T levels of the melancholic subtype were similar to the controls and, moreover, far from significantly differing from the latter (whereas the atypical depression subtype did at least on a trend-level), the clinical relevance of the elevated T levels in the current study can be questioned. Further studies are needed to replicate the findings of diverging T levels between melancholic and atypical depression with larger sample sizes.

Beyond the gonadal hormone T characterizing the atypical depression subtype, the effect size was particularly striking for BMI. In fact, it was more than twice as large as that of T. This is in line with previous findings (29, 36) demonstrating a positive association between BMI and atypical depression. A prospective, population-based study revealed that atypical depression was a strong predictor of obesity with an OR of 3.75 (62). Therefore, the BMI seems to be a constitutive characteristic of atypical depression. In the current analysis, however, the simultaneous investigation of T and BMI was not possible due to inherent model-based constraints combined with the small sample size. Further studies should determine if the low T levels of the atypical depressive subtype are simply an epiphenomenon of the high BMI associated with this subtype. Moreover, all biological systems/markers (e.g., HPA axis, HPG axis, metabolic abnormalities such as obesity, and inflammatory markers) associated with atypical depression should be balanced in their clinical relevance and better examined with regard to their possible interactions.

Taken together, the atypical depression subtype once again appeared diametrically opposed to the remaining subjects. Apart from its discriminable symptom- and comorbidity profile it also showed specific biological correlates in the current sample of men. It is remarkable that men belonging to the atypical subtype not only exhibited lower T levels but also, moreover, showed a comorbidity profile usually found in depressive women, that is to say phobias and anxiety. Further studies should show whether our speculation re a feminine profile in atypical depressive men can be confirmed.

Some studies reported a relationship between low T levels and subthreshold depressive disorders (15, 17, 20). One epidemiological study reported that lower T levels are only associated with subthreshold symptoms of anxiety and depression (19). Anxiety symptoms were not included as LCA indicators when deriving the depressive subtypes (37) and the moderate subtype including subjects with a lower severity of depression was excluded from the current analysis in order to increase statistical power. Consequently, we could not further examine this issue. However, an exploratory analysis did not indicate any deviation of the T levels in the moderate depression subgroup.

The following limitations should be noted. First, the sample sizes of the examined subsamples were small and only strong effects achieve common significance levels with small sample sizes. Although we applied robust statistical methods and additionally computed effect sizes, replications of our findings with representative, large samples are necessary. Second, we cannot draw causal conclusions from our data due to the cross-sectional design. The contradictory evidence with regard to causality of the differing T levels and affective symptoms (15, 63) needs further investigation. Hence, the current performance of regression analytic methods implying a causal direction needs to be interpreted as preliminary. Future studies are required to replicate our findings, to longitudinally analyze causal processes [particularly with regard to the interaction between HPG and HPA axes due to their great therapeutic and diagnostic potential (33)], and to assess a broader age range in order to more precisely consider the possibility of age-specific features (64–66). Third, the participants of the laboratory day were selected as a convenience sample. Consequently, a certain selection bias and a restricted external validity cannot be completely excluded. Fourth, we draw analogies between the effects of exogenous high dose T with endogenous serum T values although we can not ensure that there is a direct correlation between these biological markers. Fifth, as Jäger et al. (67) emphasized, the comparison of studies examining the issue of T deficiency is difficult because there is no international consensus on the definition of a standardized normal range of hypogonadism. Sixth, the calculation of free testosterone using total T and SHGB (68) could be added in future research.

Despite these limitations and its preliminary nature, this community study demonstrated for the first time lower serum T levels of the empirically derived atypical depression subtype in men. If the current biological findings would be replicated in larger samples, this additional differentiation of depressive subtypes by biological features may indicate distinctive pathophysiological entities and could ameliorate the specificity of depression diagnoses and treatments.

## Author Contributions

SR: designed the study, conducted all statistical analyses, and wrote the manuscript. MgH: significantly revised the manuscript. MH: significantly participated in data collection and data management. MM: significantly participated in data collection and data management. AA: significantly participated in data collection and data management of the biological parameters. WR: conceived and designed ZInEP. VA-G: conceived and designed the ZInEP Epidemiology Survey, participated in data management, and significantly revised the manuscript. All authors read and accepted the final version of the manuscript.

## Conflict of Interest Statement

The authors declare that the research was conducted in the absence of any commercial or financial relationships that could be construed as a potential conflict of interest.
